# A human-mouse conserved sex bias in amygdala gene expression related to circadian clock and energy metabolism

**DOI:** 10.1186/1756-6606-4-18

**Published:** 2011-05-04

**Authors:** Li-Chun Lin, David A Lewis, Etienne Sibille

**Affiliations:** 1 Translational Neuroscience Program, Department of Psychiatry, and Center for Neuroscience, 3811 O'Hara Street, University of Pittsburgh, Pittsburgh, PA 15213, USA

## Abstract

**Background:**

Major depression affects twice as many women as men, but the underlying molecular mechanisms responsible for the heightened female vulnerability are not known. The amygdala, composed of heterogeneous subnuclei, participates in multiple functional circuits regulating emotional responses to stress. We hypothesized that sex differences in molecular structure may contribute to differential mood regulation and disease vulnerability.

**Findings:**

Using gene arrays followed by quantitative PCR validation, we compared the transcriptome profiles between sexes in human and mouse amygdala. We now report sexually dimorphic features of transcriptomes in the basolateral nucleus of the amygdala, and these features are highly conserved across species. A functional analysis of differential gene expression showed that mitochondrial-related gene groups were identified as the top biological pathways associated with sexual dimorphism in both species.

**Conclusions:**

These results suggest that the basolateral amygdala is a sexually dimorphic structure, featuring a regulatory cascade of mitochondrial function and circadian rhythm, potentially linked through sirtuins and hormone nuclear receptors. Hence, baseline differences in amygdalar circadian regulation of cellular metabolism may contribute to sex-related differences in mood regulation and vulnerability to major depression.

## Introduction

Males and females differ in behavior and brain structure, as well as in prevalence of neuropsychiatric disorders. The greater prevalence of major depression and rapid cycling bipolar disorder in women [1,2] highlights the importance of studying potential mechanisms for sex differences. Sex chromosome- and hormone-linked genes may directly affect sexually dimorphic neurobiology [3]. For example, sex hormones regulate the size of the medial amygdala and bed nucleus of the stria terminalis during development [4]. The lateral (LA) and basal (basolateral; throughout the article we refer to human BA and mouse BLA) nuclei of the amygdala have been identified as critical sites for learned fear and emotion regulation in healthy subjects and in patients with mood disorders [5,6], but little is known about their sexual dimorphism. The isolation of subnuclei from the amygdala complex improves microarray sensitivity to detect differences in gene expression by reducing sample heterogeneity, and could facilitate the subsequent identification of genetic sex differences. To characterize sex differences in the intrinsic molecular properties of the amygdala, we examined the presence of sexually dimorphic patterns of gene expression in the basolateral nuclei of the amygdala using postmortem samples from control human subjects and mice.

## Methods

### Human Brain Samples

Postmortem amygdala samples from 12 healthy male controls and 12 healthy female controls (age = 39-64 years) were obtained from the University of Pittsburgh Brain Donation Program (Table 1), as previously described [6]. Male and female groups were matched in pairs as closely as possible on the basis of RNA integrity, pH, post-mortem interval, age, and sex, and the groups did not differ on any of these parameters (p > 0.05; Table 1). All subjects died suddenly without prolonged agonal periods. See [6] for additional details. In contrast to the light acetylcholinesterase (AChE)-stained LA across species, human BA and mouse BLA contained the heavy stained AChE-positive neuropil in the amygdala [7,8], implying that human BA and LA anatomically correspond to the mouse BLA and LA according to the well-defined AChE boundaries. Hence, based on the distribution of AChE activity and thionin staining, human lateral (LA) and basal (BA) amygdala nuclei were defined and separately harvested from 20-μm cryostat sections using a clean RNase-free pipette tip. All procedures were approved by the University of Pittsburgh's Committee for the Oversight of Research Involving the Dead and Institutional Review Board for Biomedical Research.

**Table 1:** Characteristics of matched human subjects

	Female Group						Male Group					
	
		Age		Interval				Age		Interval		
Pair	Case	(Years)	Race	(Hours)	pH	RIN	Case	(Years)	Race	(Hours)	pH	RIN
1	HU1282	39	W	24.5	6.8	7.5	HU604	39	W	19.3	7.1	8.6
2	HU1092	40	B	16.6	6.8	8.0	HU1047	43	W	12.0	6.6	9.0
3	HU627^a,b^	43	B	14.1	7.1	7.0	HU857^a,b^	48	W	16.6	6.7	8.9
4	HU567	46	W	15.0	6.8	8.9	HU1067^b^	49	W	6.0	6.6	8.2
5	HU1280^a^	50	W	23.5	6.7	7.7	HU1086^a,b^	51	W	24.2	6.8	8.1
6	HU1391^a,b^	51	W	7.8	6.6	7.1	HU852^a,b^	54	W	8.0	6.8	9.1
7	HU686	52	W	22.6	7.1	8.5	HU1031^b^	53	W	23.2	6.8	8.9
8	HU575	55	B	11.3	6.8	9.6	HU1122	55	W	15.4	6.7	7.9
9	HU1247	58	W	22.7	6.4	8.4	HU685	56	W	14.0	6.6	8.0
10	HU1318	58	W	18.8	6.7	7.4	HU852	54	W	8.0	6.8	9.1
11	HU568	60	W	9.5	6.9	8.7	HU551	61	W	16.4	6.6	8.3
12	HU1466^b^	64	B	20.0	6.7	8.8	HU615	62	W	7.2	6.4	7.8

Matched samples processed for microarray:												
	AVG	51.3		17.2	6.8	8.1		52.1		14.2	6.7	8.5
	STDEV	8.1		5.7	0.2	0.8		6.7		6.2	0.2	0.5

Matched LA for final array analysis:												
	AVG	52.4		17.9	6.8	8.4		52.4		13.5	6.7	8.4
	STDEV	8.9		5.2	0.2	0.7		7.7		5.8	0.2	0.5

Matched BA for final array analysis:												
	AVG	50.9		18.3	6.8	8.3		52.9		13.2	6.7	8.4
	STDEV	7.8		5.5	0.2	0.7		8.7		4.4	0.2	0.5

Samples from LA (marked ^a^) and BA (marked ^b^) processed but excluded due to lower microarray quality control; W = Caucasian; B = African American; RIN = RNA integrity number; AVG = average; STDVE = standard deviation.

### Mouse Brain Samples

Brains from adult C57BL/6J mice (*n *= 5 per sex; pooled by 2 from 10 mice/group, 3 months of age) were harvested, fresh frozen and cryo-sectioned at 20 μm on UV-treated laser-capture microscopy slides (Leica Microsystems, Wetzlar, Germany). Sections on the slides were dried and stored at -80°C until used. Lateral (LA) to basolateral (BLA) amygdala nuclei were visualized with rapid thionin staining and dissected with the Leica LMD 6500 laser microdissection system. All mice were maintained on a 12-hour light-dark cycle (lights on 06:00 and lights off on 18:00) with ad libitum food and water. All procedures were approved by the University of Pittsburgh Institutional Animal Care and Use Committee (protocol # 0911014A-2, Animal Assurance # A3187-01).

### RNA extraction for Microarray Samples

Total RNA was extracted using the RNeasy Plus Micro Kit (QIAGEN, Valencia, CA) and processed for microarray analysis according to manufacturer's protocol. RNA samples were amplified with Illumina TotalPrep RNA Amplification Kit (for human tissue; Illumina, San Diego, CA) and Nugen WT-Ovation Pico RNA Amplification System (for mouse tissue; NuGen, San Carlos, CA). The fragmented labeled cRNA samples were processed and hybridized to microarrays (Human HT-12v3 and MouseWG-6v2.0 BeadChips).

### Array Data Analysis

For statistical analysis, the log_2_-transformed signal intensities of probe-sets that passed the initial expression filters (< 30% missing values, detection p-value > 0.1), were extracted and renormalized to the same mean and variance across arrays in order to control for any effects of batch. Discovery threshold for male/female differences were applied in both species (two-tailed *t*-test *p*-value < 0.05; > 20% effect size). Functional analyses were performed on the identified gene list using the Ingenuity Pathways Knowledge Base [9] and compared across species.

### Real-time Quantitative PCR (qPCR) Data Analysis

Separate RNA samples were reverse-transcribed using QScript cDNA super mix (QuantaBioscience, Gaithersburg, MD) using a combination of oligo (dT) and random primers. Each PCR reaction was performed in triplicate and compared to three internal controls (GAPDH, beta-actin and cyclophilin). All designed primer sequences were designed with Primer3Plus (Additional File 1, table S1). Results were calculated as the geometric mean of relative intensities compared to the three internal controls. Validation of the array results was considered at *p*< 0.05, using one-tailed unpaired *t*-test.

## Results

**Figure 1:**
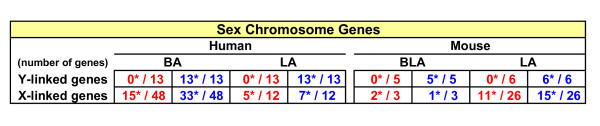
**Number of sex chromosome genes identified in the amygdala**. Red: higher expressed in females; blue: lower expressed in females. In each cell, each gene in the numerator differed significantly in expression (*p*< 0.05).

Using exploratory criteria we generated large and informative parallel human/mouse transcriptome datasets from male and female BA (BLA in mice) and LA nuclei of the amygdala. As an internal control to assess the sensitivity of the array approach, all detected Y-chromosome genes (male-specific) displayed significantly male-biased expression, and expression of numerous X-linked genes showed a female bias (Figure 1). In humans, 1335 sex-biased autosomal genes were identified in BA (Additional File 2, table S2), and fewer (*n *= 165) in LA (Additional File 3, table S3). In mice, ~1% of autosomal genes displayed sex-biased expression (BLA, *n *= 515, Additional File 4, table S4; LA, *n *= 556, Additional File 5, table S5).

To reduce false discovery and focus on the most relevant changes, we characterized the functional and cross-species relatedness of autosomal sex-biased gene sets using the Ingenuity Pathways Analysis (IPA) database. Mitochondrial-related functions were consistently identified among the top four most-represented pathways in human BA (*p*< 1.57E^-06^), mouse BA (*p*< 8.37E^-03^) and mouse LA (*p*< 6.72E^-02^). Assembly of RNA Polymerase III Complex was the only identified canonical pathway in human LA and was at trend level (*p *< 0.1).

**Figure 2:**
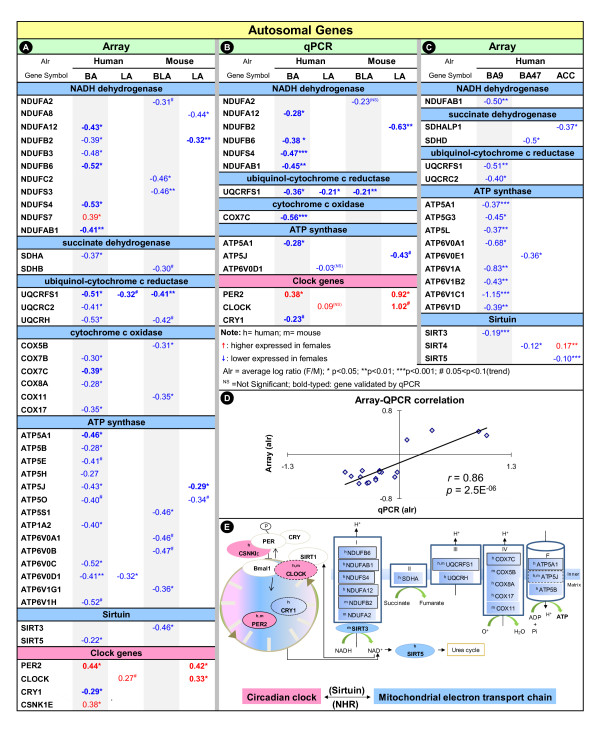
**Human-mouse conserved sexually dimorphic gene expression in the amygdala**. Sexually dimorphic gene expression in human BA/LA and mouse BLA/LA nuclei by microarray (A) and qPCR (B) analysis. (C) Sexually dimorphic gene expression in human BA9 and BA47. ALR indicates female to male expression ratios (in log_2_). (D) Correlation summary graph of array/qPCR confirmation of differential gene expression. (E) Schematized network of male-biased (blue) mitochondrial genes and female-biased (red) clock genes. Continuous contour indicates 12 sex-biased genes confirmed by qPCR (*p*< 0.05). Dashed-lined contour indicates qPCR results at the trend level (*p*< 0.1). NHR = nuclear hormone receptor.

Within the mitochondrial-related functions, genes encoding enzymes of the mitochondrial electron transport chain (ETC) consistently displayed high male-biased expression (Figure 2A). Also, two genes coding for mitochondria-localized sirtuins (SIRT3, SIRT5), which serve as metabolic regulators of circadian rhythms by utilizing NAD^+ ^[10], also showed similar high male-biased expression in human BA and mouse BLA. Of related interest was the identification of sex-biased circadian rhythm signaling in human BA (IPA, *p*< 5.39E^-04^) and mouse LA (*p*< 3.54E^-02^), which included conserved high female-biased expression of selected circadian clock genes (e.g., Period 2 and trend-level for CLOCK; Figure 2A). Intriguingly, as nuclear receptors link the circadian clock to metabolism [11], retinoic acid orphan receptor β (RORB) and thyroid hormone receptor α (THRA) displayed high female-biased expression in human BA (RORB: average log ratio [alr] = 0.29, *p *< 0.026; THRA: alr = 0.31, *p *< 0.013).

The accuracy and reproducibility of the approach was assessed by qPCR on independent samples. 12 genes out of 18 tested were individually confirmed (*p*< 0.05; same direction; Figure 2B). Overall, the qPCR validation results were highly correlated with array data using Pearson tests for significance (*r *= 0.86, *p *< 2.5E^-06^; Figure 2D).

We next evaluated the anatomical extent of the observed sex-biased gene profiles in other brain areas using existing human array data in cortical regions [12]. Numerous ETC and SIRT genes in dorsolateral prefrontal cortex (Brodman area 9 [BA9]; Figure 2C), and fewer in the ventrolateral prefrontal cortex (Brodman area 47 [BA47]; Figure 2C) and anterior cingulate cortex (ACC; Figure 2C), consistently displayed high male-biased expression, suggesting variable levels of regional conservation and amygdala enrichment.

Notably, within the limited cohort size and age range, we did not observe statistically significant age-related effects for genes confirmed by qPCR, except for male-biased ATP synthase genes (ATP5A1, *r *= -0.78, *p *< 0.005; ATP6VOD1, *r *= -0.58, *p *< 0.05) in human male amygdala. Since no information was available for menopause or estrogen replacement treatment for the human female subjects, we interpreted these results as indicative of sexual dimorphism independently of age or estrogen status, although this will need to be tested in larger cohorts.

## Discussion

We report sexually dimorphic gene expression profiles in the BA nucleus of the amygdala in human subjects. Changes at the level of single genes extend to homologous BLA nucleus in the mouse amygdala, albeit to a lesser extent. At the functional level, mitochondrial-related gene groups were consistently identified as the top biological pathways associated with male/female differences in both species. Together with prominent differences in other gene groups (Figure 2), our results suggest a sexual dimorphism in the expression level of an evolutionarily conserved pathway - circadian clock and mitochondrial function, potentially linked through sirtuin and nuclear hormone receptors.

In experimental models, circadian genes modulate energy metabolism by setting the periodic oscillations of expression and activity of enzymes to influence physiological and behavioral functions. Substantial evidence has shown that patients with major depression or bipolar disorder often display abnormalities in circadian rhythm, such as disturbances in the sleep/wake cycle and diurnal mood changes. Genetics studies have found associations with CLOCK, PER1-3, and CRY1, and Sirtuin genes in mood disorders [13-16]. In molecular/genetic studies, mitochondrial dysfunction has been associated with mood disorders [17,18], and mood disorders are common in patients with mitochondrial disorders [19]. Thus, our findings suggest that baseline sexual dimorphism in mitochondrial density or functions may influence energy availability in the amygdala. Notably, while mitochondrial function was systemically identified as a top sex-biased gene family across species, the species-specific orthologous genes were not necessarily and systematically the same, but very close functionally (Figure 2E). Because of identification of mitochondrial-related genes with closely related functions and locations across species, we speculate that the sex-biased mitochondria profile may be under conserved transcription control to display similar sexual dimorphism across species.

Limitations of this study are noted. The results will need to be confirmed at the protein and functional levels in independent and larger cohorts. Similarly, the potential contribution of race-specific amygdala gene expression will need to be assessed in larger cohorts. It is also unclear whether our findings in mid-life adults can be generalized to adolescent and elderly populations. Here we identified age effects in 2 of the 18 genes validated by qPCR, suggesting that aging may not contribute to the evolutionary conserved genetic sex differences in amygdala. In addition, this study sampled 10 intact female mice and 12 female human subjects from 39-64 years old, which should considerably lower the impact of variable estrogen levels on our findings of genetic sex difference, although we cannot exclude residual effects.

In conclusion, we present evidence for significant male/female differences in amygdala gene expression across species. Delineating the contribution of mitochondrial and circadian signaling in mood disorders will require additional work. For instance, sex-biased gene expression of circadian clock, nuclear hormone receptors, mitochondrial sirtuins and electron chain enzymes in an interlocking-feedback loop, may functionally translate into differential amygdala responsiveness (Figure 2E). This loop may lead to sex differences in metabolic capacity to meet energy demand, and might potentially contribute to the greater female vulnerability to mood disorders.

## List of abbreviations used

ACC: anterior cingulate cortex; AChE: acetylcholinesterase; BA: basal amygdala; BA9: Brodman area 9; BA47: Brodman area 47; BLA: basolateral amygdala; ETC: electron transport chain; IPA: Ingenuity Pathways Analysis; LA: lateral amygdala; qPCR: Real-time Quantitative PCR; RORB: retinoic acid orphan receptor β; SIRT: sirtuin; THRA: thyroid hormone receptor α.

## Competing interests

Dr. Lewis currently receives investigator-initiated research support from the BMS Foundation, Bristol-Myers Squibb, Curridium Ltd and Pfizer and in 2007-2010 served as a consultant in the areas of target identification and validation and new compound development to AstraZeneca, BioLine RX, Bristol-Myers Squibb, Hoffman-Roche, Lilly, Merck, Neurogen, and SK Life Science.

## Authors' contributions

LCL and ES designed this study. LCL carried out the molecular experiments, performed the statistical analysis and drafted the manuscript. DAL provided technical and conceptual support for the human brain collection. The final manuscript was written by LCL and ES. All authors have read, edited and approved the final manuscript.

## Supplementary Material

Additional file 1 **Table S1. Sequence of human and mouse primers used for qPCR measurements**. Click here for file

Additional file 2 **Table S2. Sex-biased genes in human BA**. Sex-biased genes detected by the microarray analysis in the postmortem basal amygdala samples from healthy human subjects. Click here for file

Additional file 3 **Table S3. Sex-biased genes in human LA**. Sex-biased genes detected by the microarray analysis in the postmortem lateral amygdala samples from healthy human subjects. Click here for file

Additional file 4 **Table S4. Sex-biased genes in mouse BLA**. Sex-biased genes detected by the microarray analysis in the basolateral amygdala from adult C57BL/6J mice. Click here for file

Additional file 5 **Table S5. Sex-biased genes in mouse LA**. Sex-biased genes detected by the microarray analysis in the lateral amygdala from adult C57BL/6J mice. Click here for file
