# Supplementary material for: A human-mouse conserved sex bias in amygdala gene expression related to circadian clock and energy metabolism
Source: Mol Brain. 2011 May 4;4:18. doi: 10.1186/1756-6606-4-18 (PMC3098780; doi:10.1186/1756-6606-4-18)
Supplement: Additional file 1 — Table S1. Sequence of human and mouse primers used for qPCR measurements. [file 1756-6606-4-18-S1.DOC]

**Table S1. Sequence of human and mouse primers used for qPCR measurements.**

| **Gene** | **Species** | **Accession No.** | **5' sequence** | **3' sequence** |
| --- | --- | --- | --- | --- |
| NDUFA12 | Human | NM_018838.3 | GGACAAATGATGCGAAGGTT | TACTGTGAAGCCAACGATGC |
| NDUFB6 | Human | NM_002493.3 | CACTCCGGATGAGAAACTGC | CATGGACCATTTTCCTCCAA |
| NDUFS4 | Human | NM_002495.1 | AGAGCGATGGGAAAATCCTT | CTTGGACTTGGGTTTTGGAA |
| NDUFAB1 | Human | NM_005003.2 | CATCCAGGACCGTGTTCTTT | CAAACCCAAATTCGTCTTCC |
| UQCRFS1 | Human | NM_006003.1 | GGAAATTGAGCAGGAAGCTG | GGCAAGGGCAGTAATAACCA |
| COX7C | Human | NM_001867.2 | CCGTAGGAGCCACTATGAGG | GGCTGCACCTCTTAAAATGC |
| ATP5A1 | Human | NM_004046.4 | CATTGTGGACGTTCCAGTTG | ATTGGCACCAAGCTATCCAC |
| ATP6V0D1 | Human | NM_004691.3 | CCTGGAGTTTGAAGCAGACC | CTACCTGCACCCTCGAAGAG |
| PER2 | Human | NM_022817.1 | CTGGCCATCCACAAAAAGAT | CCTCCCAATGATGAAGGAGA |
| CLOCK | Human | NM_004898.2 | GGCTGAAAGACGACGAGAAC | GGTGTTGAGGAAGGGTCTGA |
| CRY1 | Human | NM_004075.2 | TTGCTTGATGCAGATTGGAG | TTTTGCAGGGAAGCCTCTTA |
| GAPDH | Human | NM_002046.3 | TGCACCACCAACTGCTTAGC | GGCATGGACTGTGGTCATG |
| CYCLO | Human | NM_021130.3 | GCAGACAAGGTCCCAAAG | GAAGTCACCACCCTGACAC |
| ACTIN | Human | NM_001101.3 | ACGGTGAAGGTGACAGCA | TTAGGATGGCAAGGGACTTC |
| NDUFA2 | Mouse | NM_010885.4 | GGTGTGAGGGATTTCATCGT | CTGCATGGCTCTGGTTACCT |
| NDUFB2 | Mouse | NM_026612.1 | ACATATTCAGCCCCGGTACA | CGAGGGATTCCCAGTTCTTC |
| UQCRFS1 | Mouse | NM_025710.1 | TGGTCTCCCAGTTTGTTTCC | GCAGCTTCCTGGTCAATCTC |
| ATP5J | Mouse | NM_016755.2 | GTCCTTCGGTCAGCAGTCTC | CTGGGCCAATATCAACAGGT |
| PER2 | Mouse | NM_011066.1 | TGCTTACACGGGTGTCCTAA | CTGGGTGAAGGTACGTTTGG |
| CLOCK | Mouse | NM_007715.5 | AAGACCATTCCCCAAGCTCT | GCTTCAGTGCTCCCAACTTC |
| GAPDH | Mouse | NM_008084.2 | AACTCCCACTCTTCCACCT | CACCACCCTGTTGCTGTA |
| CYCLO | Mouse | NM_008907.1 | AAAGCCGATGACAAGGAG | AAGAATCGTCGCTGGTATG |
| ACTIN | Mouse | NM_007393.3 | CCTAGCACCATGAAGATCAA | GGAAGGTGGACAGTGAGG |
